# Association between dietary fat intake and fatty acid profiles and hyperuricemia among Chinese adults: Results from the China Health and Nutrition Survey

**DOI:** 10.6133/apjcn.202604_35(2).0007

**Published:** 2026-03-17

**Authors:** Pengfeng Qu, Yingying Jiao, Liusen Wang, Weiyi Li, Hongru Jiang, Jiguo Zhang, Huijun Wang, Bing Zhang, Junhua Han, Aidong Liu, Zhihong Wang

**Affiliations:** 1 Office of National Nutrition Plan, National Institute for Nutrition and Health, Chinese Center for Disease Control and Prevention, Beijing, China; 2 Hancheng Center for Disease Control and Prevention, Shaanxi, China; 3 Key Laboratory of Trace Elements and Nutrition, National Health Commission, Beijing, China; 4 Chinese Nutrition Society, Beijing, China

**Keywords:** fats, saturated fatty acids, monounsaturated fatty acids, polyunsaturated fatty acids, hyperuricemia

## Abstract

**Background and Objectives:**

Diet is a modifiable factor influencing serum uric acid levels, but evidence on the associations between dietary fat composition and hyperuricemia (HUA) remains limited. This study examined the relationships between the proportion of energy from total fat and specific dietary fatty acids and the risk of HUA among Chinese adults.

**Methods and Study Design:**

Data were obtained from adults who participated in at least two follow‑up waves of the China Health and Nutrition Survey (CHNS) in 2009, 2015, and 2018. Associations and dose–response relationships were assessed using multivariate Cox proportional hazards and restricted cubic spline (RCS) models.

**Results:**

During a mean follow‑up of 6.05 years among 2,722 participants, the prevalence of HUA was 10.2% (men: 14.1%; women: 7.76%). In women, energy from saturated fatty acids (SFAs) in the fourth quintile (7.88%) and from monounsaturated fatty acids (MUFAs) in the third quintile (9.97%) was positively associated with HUA (HR = 2.19, 95% CI 1.19-4.05; HR = 2.14, 95% CI 1.21-3.79), whereas polyunsaturated fatty acids (PUFAs) in the third quintile (6.88%) were negatively associated (HR = 0.41, 95% CI 0.22-0.78). RCS analyses showed J‑shaped and U‑shaped associations in men between total fat (37.9%, 95% CI 11.0-40.3) and PUFAs energy ratio (9.60%, 95% CI 2.25-10.7) with HUA, respectively, and an L‑shaped association in women for PUFAs energy ratio (6.25%, 95% CI 5.54-9.42).

**Conclusions:**

These findings suggest sex‑specific, non‑linear relationships between total fat and different fatty acid intakes and HUA risk. Men should consider moderating total fat intake, while women should limit SFAs and moderately increase PUFAs to help reduce HUA risk.

## INTRODUCTION

Hyperuricemia (HUA) is a prevalent metabolic disease and a precursor to gout.^1^ It is also associated with an elevated risk of diabetes, chronic kidney disease, cardiovascular diseases, and metabolic syndrome. The prevalence of HUA among Chinese adults increased from 11.1% in 2015-2016 to 14.0% in 2018-2019,^2^ highlighting the urgency of identifying modifiable risk factors.

The pathogenesis of HUA is complex, encompassing a multitude of factors including genetics,^3^ diet,^4^ and lifestyle.^5^ Although genetics contributes to the development of HUA, dietary interventions present a crucial strategy for its prevention and management.^5^ Fats are essential macronutrients in the diet, and the dietary fat energy ratio and different subtypes of fatty acids have distinct impacts on health.^6-9^ Several studies have indicated an association between dietary fat and HUA.^10-12^ However, these studies have primarily focused on specific populations or dietary interventions with supplements, rather than examining the daily diet of the general population. Moreover, the differential effects of various types of dietary fat on HUA have not been fully explored.

In light of these considerations, we aim to investigate the potential associations between dietary fat intake, particularly the intake of saturated fatty acids (SFAs), monounsaturated fatty acids (MUFAs), and polyunsaturated fatty acids (PUFAs), and the risk of HUA among Chinese adults. We propose that a diet high in fat, especially one rich in SFAs, may trigger a series of physiological alterations that enhance the risk of HUA. These changes include insulin resistance, which is associated with reduced uric acid clearance by the kidneys and elevated serum uric acid (SUA) levels;^13,14^ dyslipidemia, which can promote hepatic urate biosynthesis and increase SUA levels;^15,16^ chronic inflammation, which may be implicated, can be induced by SFAs that elicit Toll-like receptor-mediated inflammatory responses,^17^ leading to elevated levels of inflammatory biomarkers, and the establishment of a chronic inflammatory condition that may potentiate the risk of HUA via multiple pathways.^18,19^ Conversely, PUFAs are recognized for their anti-inflammatory properties, which may help to alleviate inflammation and are associated with a reduced risk of HUA.^20-23^

This study seeks to clarify how the proportion of total energy intake derived from fats, together with the consumption of specific fatty acids, is associated with the risk of HUA among Chinese adults. Using a prospective design within a substantial population-based cohort, we adjusted for extensive confounding variables and applied rigorous statistical methodologies to analysis these relationships. By doing so, we aim to provide more robust evidence for the prevention and management of HUA.

## METHODS

### Study participants

This study employs data from the China Health and Nutrition Survey (CHNS), a comprehensive and multifaceted longitudinal cohort study designed to evaluate trends in health and nutrition status and investigate the underlying causes of nutritional disorders among the Chinese population.^24^ Initiated in 1989, the CHNS has conducted follow-ups at intervals of 2–4 years and, by 2018, encompassed fifteen provinces that differ in demographics, geography, economic growth, and availability of public resources.^25^ A multi-stage random cluster sampling method was employed to select participants in each province, with the detailed sampling procedures described in other publications.^26^ Data for this analysis were derived from three cycles (2009, 2015, and 2018) of the CHNS, each of which included the collection of fasting venous blood samples. The study population comprised adults aged between 18 and 59 years who had comprehensive data on dietary intake and SUA levels. Exclusion criteria were applied to individuals who were pregnant or breastfeeding, those with incomplete survey responses, and those who reported unusually high or low energy consumption (for men: greater than 6000 kcal or less than 800 kcal; for women: greater than 4000 kcal or less than 600 kcal). Furthermore, participants with a body mass index (BMI) outside the 14 to 45 kg/m² range, as well as those with a prior history of stroke, cancer, or baseline HUA, were removed from the analysis. Individuals who had only a single follow-up assessment were excluded as well. In total, 3657 individuals were retained for the ultimate analysis. The process of participant selection is illustrated in Supplementary Figure 1.

The research adhered to the principles of the Declaration of Helsinki, with the study protocol receiving approval from the Ethics Committee of the University of North Carolina at Chapel Hill (Project ID: 07-1963) and the National Institute for Nutrition and Health, Chinese Center for Disease Control and Prevention (Project ID: 2018-004). Approval was granted on March 14, 2018. Before participating in the research, all individuals gave their voluntary consent after being fully informed.

### Measurement of HUA

Following an 8-to-12-hour overnight fast, participants provided blood samples, which were collected, processed, and stored by trained personnel according to standardized protocols. The fasting blood samples were then analyzed at a central laboratory in Beijing under rigorous quality assurance procedures. The primary endpoint, serum uric acid concentration, was quantified in µmol/L using a colorimetric enzymatic method. HUA was defined as SUA ≥ 7.0 mg/dL (420 μmol/L) in males and ≥6.0 mg/dL (360 µmol/L) in females.^27^

### Measurement of dietary fat intake and fatty acid profile

The assessment of dietary consumption was conducted using a 3-day consecutive 24-hour recall method, which included two weekdays and one weekend day, at the personal level. Participants were asked to provide a detailed account of the kinds and quantities of food and drinks they had consumed during the last 24 hours.^28^ Interviewers received training to utilize standardized forms, food models, and picture aids when conducting 24-hour dietary recalls in household interviews. Simultaneously, the use of cooking oil and condiments was documented over a 3-day period through household weighing, and this data was apportioned to individuals according to their share of total energy intake. The Chinese Food Composition Table (CFCS) was then employed to convert food and condiment consumption into corresponding energy and nutrient intake values. Ultimately, the average daily intake of energy, fat, and specific fatty acids (SFAs, MUFAs, and PUFAs) per person was determined based on the total person-days recorded throughout the duration of the data collection phase.

In our study, dietary data tracking was stopped once participants were classified as having HUA to avoid bias from subsequent nutritional modifications. Data handling was as follows: For participants who began in 2009 and were classified with HUA in 2015, we used the 2009 dietary data. For those classified in 2018, we used the average of their 2009 and 2015 dietary data. For those who enrolled in 2015 and were classified with HUA in 2018, we used the 2015 dietary data.

### Covariates

The following variables were assessed as potential covariates: age (18-49 years, 49-59 years); sex (male, female); education attainment (grades 1-6 or lower, junior or senior high school, university level or higher); household income per capita (tertiles: low [<5618 CNY], medium [5618-14000 CNY], high [>14000 CNY]); geographic region (eastern, central, and western); residential area (urban or rural); smoking status (current non-smoker and current smoker); and alcohol intake (non-drinker in the past year and current drinker). The level of physical activity was measured in terms of metabolic-equivalent hours per week (MET-h/week) and categorized into three groups (low, moderate, high) based on the intensity of activities.^29^ Total energy intake was treated as a continuous variable. Total purine intake as a continuous variable. BMI was computed from height and weight measurements and is considered a continuous variable. Values of estimated glomerular filtration rate (eGFR: mL/min/1.73 m^2^) were calculated using the equation proposed by investigators in the Chronic Kidney Disease Epidemiology Collaboration and treated as a continuous variable.^30^

### Statistical analysis

Categorical variables were presented as proportions, whereas continuous variables with non-normal distribution are characterized using medians (Q1, Q3). Kruskal-Wallis was used for comparisons of baseline variables with continuous non-normal distributions. Categorical variables were tested using chi-square tests. Individuals were categorized into quintiles according to the energy contribution from fats, SFAs, MUFAs, and PUFAs.

The associations between the energy contribution from fats and the intake of SFAs, MUFAs, and PUFAs were evaluated using Cox proportional hazards regression models. Model 1 was adjusted for age, education, per capita family income, geographic region, and residential area. Model 2 additionally adjusted for the degree of physical activity, total energy intake, total purine intake. Model 3 was further adjusted for eGFR and BMI for continuous variables. HR with 95% CI were calculated accordingly. The median values of the proportion of energy intake from fats, as well as the intakes of SFAs, MUFAs, and PUFAs, were used as continuous variables to assess trends within the model.

Further, we generated restricted cubic spline (RCS) plots using Cox proportional hazards models with four knots to explore the nonlinear dose-response relationship between exposure variables and HUA.^31^

All data were cleaned, managed, and analyzed using SAS 9.4 (SAS Institute, Inc., Cary, NC, USA) as well as R software, version 4.1.0 (The R Foundation for Statistical Computing). A *p*-value of less than 0.05 for a two-tailed test was considered to indicate statistical significance in all analyses.

## RESULTS

### Baseline characteristics

The study had an average follow-up period of 6.05 years (standard deviation 2.62), and the overall incidence of HUA was 10.2% (278/2722), with a prevalence of 14.1% (148/1,047) in men and 7.76% (130/1675) in women. Table 1 presents the distribution of participant characteristics across quintiles of fat energy intake in males and females. Participants in the lowest quintile, regardless of sex, tended to have lower educational attainment, lower household income, and rural residence. In contrast, those in the highest quintile, both men and women, exhibited higher total energy and purine intakes. Gender‑specific trends were also evident: men in the top quintile were more likely to have higher BMI and lower physical activity levels, whereas women in this quintile reported a modestly higher frequency of alcohol drinking, although the overall prevalence remained low.

### Association of the proportion of energy intake from fats with HUA

Table 2 presents the association between quintiles of the proportion of energy intake from fats and HUA in men and women. No statistically significant associations were observed in either sex after adjustment for demographic, lifestyle, dietary, and renal function factors.

### Association of dietary fatty acids intake with HUA

Table 3 presents the relationship between SFAS intake and HUA risk in men and women. Among women, a positive association was found between SFAS intake and HUA. After initial adjustment (Model 1), the HR for the fourth quintile compared to the lowest was 2.27 (95% CI 1.24–4.17). This association remained significant after further adjustment for lifestyle factors and dietary intake (Model 2: HR = 2.35; 95% CI 1.28–4.33). In the final model, additionally adjusted for BMI and eGFR, the association persisted for the fourth quintile (HR = 2.19; 95% CI 1.19–4.05) but was not significant for the fifth quintile (HR = 1.54; 95% CI 0.80–2.98). The overall trend across quintiles was not statistically significant (p for trend = 0.13). No statistically significant association was observed between SFAS intake and HUA among men after multivariable adjustment.

Table 4 shows the relationship between MUFAs intake and HUA risk in men and women. Among women, a significant positive association was consistently observed between MUFAs intake and HUA in the Q3 across all adjustment models. The HRs (95% CIs) were 1.82 (1.03–3.21) in Model 1, 1.84 (1.04–3.24) in Model 2, and 2.14 (1.21–3.79) in the fully adjusted Model 3. Associations for the other quintiles were not statistically significant, including the highest quintile (HR = 1.09; 95% CI 0.58–2.06 in Model 3). The overall trend across quintiles was not statistically significant (*p* for trend = 0.54). No statistically significant association was observed between MUFAs intake and the risk of HUA among men.

Table 5 shows the relationship between PUFAs intake and HUA risk in men and women. Among women, a significant inverse association was consistently observed between PUFAs intake and HUA in the Q3 across all adjustment models. The HR (95% CIs) were 0.36 (0.19–0.67) in Model 1, 0.36 (0.19–0.68) in Model 2, and 0.41 (0.22–0.78) in the fully adjusted Model 3. Associations for the other quintiles were not statistically significant, including the highest quintile (HR = 0.94; 95% CI 0.57–1.55 in Model 3). The overall trend across quintiles was not statistically significant (*p* for trend = 0.87). Among men, PUFAs intake was not significantly associated with HUA risk.

**Table 1:** Baseline characteristics of participants by quintile of proportion of energy intake from dietary fats in males and females (n= 2722)^†^

Characteristic	Quintile of proportion of energy intake from fats											
	Male						Female					
	Q1 (n=209)	Q2 (n=210)	Q3 (n=209)	Q4 (n=210)	Q5 (n=209)	*p*-value^‡^	Q1 (n=335)	Q2 (n=335)	Q3 (n=335)	Q4 (n=335)	Q5 (n=335)	*p*-value^‡^
Age/ (%)						0.566						0.241
18~49	82.3	77.6	80.4	78.6	76.1		83.3	85.1	84.5	84.2	79.1	
49~59	17.7	22.4	19.6	21.4	23.9		16.7	14.9	15.5	15.8	20.9	
Education/ (%)						<0.001						<0.001
Low	22.5	20.0	15.3	18.1	12.4		38.2	32.5	26.3	23.9	25.4	
Medium	55.5	37.1	45.0	40.5	39.2		41.5	38.2	37.3	40.0	36.7	
High	22.0	42.9	39.7	41.4	48.3		20.3	29.3	36.4	36.1	37.9	
Household income/ (%)						0.147						0.001
Low	39.2	36.7	32.1	27.1	27.7		42.7	35.5	30.2	27.8	32.8	
Medium	31.1	30.0	31.6	35.7	32.5		32.2	31.6	31.9	34.0	31.0	
High	29.7	33.3	36.4	37.1	39.7		25.1	32.8	37.9	38.2	36.1	
Geographic region/ (%)						0.372						<0.001
Eastern	32.5	38.1	34.5	37.6	36.8		32.5	43.9	39.7	40.6	32.8	
Central	46.9	36.2	41.6	40.5	35.4		52.8	34.9	39.4	36.7	33.7	
Western	20.6	25.7	23.9	21.9	27.8		14.6	21.2	20.9	22.7	33.4	
Urban/ (%)	21.1	25.7	30.6	33.8	43.1	<0.001	22.1	25.4	32.5	36.1	46.6	<0.001
Current non-smoker/ (%)	40.2	38.6	39.2	42.9	47.4	0.350	97.3	97.6	97.0	98.2	97.9	0.865
Non-drinker in the past year/ (%)	36.4	40.0	36.4	39.5	38.8	0.899	94.3	93.1	91.0	90.8	87.8	0.029
Physical activity/ (%)						0.014						0.065
Low	28.2	29.1	24.9	31.0	31.1		26.0	25.7	28.4	29.3	34.9	
Medium	26.3	31.4	41.6	35.7	38.8		34.0	38.2	38.2	38.2	36.4	
High	45.5	39.5	33.5	33.3	30.1		40.0	36.1	33.4	32.5	28.7	
BMI / (kg/m^2^)	22.5	22.2	23.1	23.2	23.4	0.010	23.1	22.8	22.8	22.6	23.1	0.398
	(3.93)	(4.31)	(4.12)	(4.31)	(4.61)		(3.82)	(4.34)	(4.28)	(3.87)	(4.35)	
Total energy intake (kcal/d)	2277	2517	2465	2466	2397	0.022	1894	1953	1940	1980	2042	0.065
	(901)	(802)	(1024)	(817)	(910)		(909)	(743)	(646)	(721)	(795)	
Fat/%E	19.9	27.8	32.9	38.1	46.4	<0.001	19.5	27.6	32.9	38.6	46.8	<0.001
(6.33)	(3.66)	(2.32)	(3.19)	(6.14)		(6.41)	(2.79)	(2.89)	(2.97)	(7.59)	
Total purine intake (mg/d)	152	173	175	187	189	<0.001	156	177	185	193	194	<0.001
(77.1)	(78.9)	(84.1)	(89.6)	(105)		(86.0)	(83.2)	(89.5)	(88.8)	(93.6)	
eGFR (mL/min/1.73 m^2^)	94.5	91.8	90.7	90.6	89.6	0.023	87.9	87.2	87.4	89.3	86.0	0.040
	(18.5)	(20.0)	(19.8)	(18.4)	(17.5)		(17.7)	(20.4)	(16.3)	(19.5)	(16.5)	

BMI, body mass index; eGFR, estimated glomerular filtration rate.

† Categorical variables were reported as n (%); BMI and total energy intake were reported as median (IQR); Fat/%E was reported as median (IQR). Total purine intake is presented as the median (IQR), and eGFR is presented as the median (IQR) .

‡ *p*-values were calculated by Kruskal-Wallis for continuous variables, and χ2 test for categorical variables.

**Table 2:** HR and 95% CI for the association between the proportion of energy intake from fats and HUA risk in male and female^†^^‡^

	Quintile of proportion of energy intake from fats					*p*-trend
	Q1	Q2	Q3	Q4	Q5	
Male						
Fat /%E M (Q_1_, Q_3_)/	21.8 (18.7, 24.3)	29.4 (27.9, 30.5)	34.0 (32.8, 35.3)	38.9 (37.6, 40.3)	46.2 (44.0, 50.1)	
Model 1^§^	1 (ref)	0.87 (0.52, 1.45)	0.71 (0.42, 1.22)	0.65 (0.38, 1.12)	1.27 (0.79, 2.06)	0.410
Model 2^¶^	1 (ref)	0.88 (0.53, 1.48)	0.74 (0.43, 1.26)	0.68 (0.40, 1.17)	1.27 (0.78, 2.06)	0.391
Model 3^††^	1 (ref)	0.75 (0.45, 1.26)	0.60 (0.39, 1.15)	0.65 (0.38, 1.11)	0.87 (0.54, 1.42)	0.707
Female						
Fat/%E M (Q_1_, Q_3_)/	22.1 (18.6, 24.2)	29.4 (27.8, 30.8)	34.3 (33.2, 35.5)	39.5 (38.0, 40.7)	47.3(44.8, 51.6)	
Model 1^§^	1 (ref)	1.29 (0.73, 2.31)	1.19 (0.67, 2.14)	0.72 (0.37, 1.39)	1.48 (0.83, 2.67)	0.536
Model 2^¶^	1 (ref)	1.34 (0.75, 2.40)	1.23 (0.68, 2.23)	0.72 (0.37, 1.40)	1.56 (0.86, 2.83)	0.478
Model 3^††^	1 (ref)	1.35 (0.76, 2.43)	1.39 (0.77, 2.50)	0.67 (0.34, 1.30)	1.31 (0.73, 2.37)	0.976

HUA, hyperuricemia.

† Continuous variables were reported as median (Q1, Q3).

‡ The Cox proportional hazards model was used to estimate hazard ratios.

§ Model 1 adjusted for age, education, per-capita household income, geographic region, and place of residence.

¶ Model 2 additionally adjusted for physical activity level, total energy intake (continuous), and total purine intake (continuous).

†† Model 3 further adjusted for estimated glomerular filtration rate (eGFR, continuous) and BMI (continuous).

* *p* < 0.05.

**Table 3:** HR and 95% CI for the association between SFAs intake and HUA in males and females^†^^‡^

	Quintile of proportion of energy intake from fats					*p*-trend
	Q1	Q2	Q3	Q4	Q5	
Male						
SFAs / %E M (Q_1_, Q_3_)	3.58 (2.85, 4.18)	5.27 (4.97, 5.65)	6.56 (6.24, 6.85)	7.83 (7.36, 8.25)	10.1 (9.43, 11.6)	
Model 1^§^	1 (ref)	1.14 (0.67, 1.93)	0.85 (0.48, 1.51)	1.04 (0.59, 1.81)	1.47 (0.87, 2.50)	0.162
Model 2^¶^	1 (ref)	1.05 (0.62, 1.80)	0.84 (0.48, 1.50)	1.02 (0.58, 1.79)	1.42 (0.83, 2.43)	0.166
Model 3^††^	1 (ref)	1.18 (0.69, 2.02)	0.91 (0.51, 1.61)	0.83 (0.47, 1.46)	1.20 (0.71, 2.03)	0.732
Female						
SFAs / %E M (Q_1_, Q_3_)	3.55 (2.82, 4.02)	5.29 (4.94, 5.63)	6.63 (6.30, 6.91)	7.88 (7.53, 8.34)	10.1 (9.31, 11.2)	
Model 1^§^	1 (ref)	1.47 (0.77, 2.81)	1.22 (0.63, 2.36)	2.27** (1.24, 4.17)	1.40 (0.73, 2.70)	0.200
Model 2^¶^	1 (ref)	1.47 (0.77, 2.82)	1.24 (0.64, 2.40)	2.35** (1.28, 4.33)	1.44 (0.74, 2.78)	0.167
Model 3^††^	1 (ref)	1.44 (0.75, 2.77)	1.42 (0.73, 2.76)	2.19* (1.19, 4.05)	1.54 (0.80, 2.98)	0.128

HUA, hyperuricemia; SFAs, saturated fatty acids.

† Continuous variables were reported as median (Q1, Q3).

‡ The Cox proportional hazards model was used to estimate hazard ratios.

§ Model 1 adjusted for age, education, per-capita household income, geographic region, and place of residence.

¶ Model 2 additionally adjusted for physical activity level, total energy intake (continuous), and total purine intake (continuous).

†† Model 3 further adjusted for estimated glomerular filtration rate (eGFR, continuous) and BMI (continuous).

* *p* < 0.05,

** *p* < 0.01

**Table 4:** HR and 95% CI for the association between MUFAs intake and HUA in males and females^†^^‡^

	Quintile of proportion of energy intake from fats					*p*-trend
	Q1	Q2	Q3	Q4	Q5	
Male						
MUFAs / %E M (Q_1_, Q_3_)	4.96 (3.93, 5.97)	7.77 (7.11, 8.28)	10.2 (9.66,10.7)	12.9 (12.2, 13.6)	17.7 (15.9, 19.4)	
Model 1^§^	1 (ref)	1.05 (0.63,1.75)	1.06 (0.63,1.79)	0.71 (0.40, 1.25)	1.05 (0.62, 1.76)	0.802
Model 2^¶^	1 (ref)	0.99 (0.59,1.67)	1.05 (0.62,1.78)	0.74 (0.42, 1.30)	1.02 (0.61, 1.73)	0.834
Model 3^††^	1 (ref)	1.09 (0.65,1.82)	0.93 (0.55,1.57)	0.69 (0.39, 1.23)	0.91 (0.54, 1.54)	0.419
Female						
MUFAs / %E M (Q_1_, Q_3_)	5.09 (3.95, 6.01)	7.85 (7.36, 8.36)	9.97 (9.39, 10.6)	13.00 (12.16, 0.83)	17.7 (16.2, 20.6)	
Model 1^§^	1 (ref)	1.28 (0.70,2.33)	1.82* (1.03, 3.21)	0.91 (0.48, 1.74)	1.07 (0.57, 2.01)	0.538
Model 2^¶^	1 (ref)	1.27 (0.70,2.32)	1.84* (1.04, 3.24)	0.93 (0.48, 1.78)	1.09 (0.57, 2.07)	0.605
Model 3^††^	1 (ref)	1.37 (0.75,2.50)	2.14* (1.21, 3.79)	1.01 (0.53, 1.95)	1.09 (0.58, 2.06)	0.540

HUA, hyperuricemia; MUFAs, monounsaturated fatty acids.

† Continuous variables were reported as median (Q1, Q3).

‡ The Cox proportional hazards model was used to estimate hazard ratios.

§ Model 1 adjusted for age, education, per-capita household income, geographic region, and place of residence.

¶ Model 2 additionally adjusted for physical activity level, total energy intake (continuous), and total purine intake (continuous).

†† Model 3 further adjusted for estimated glomerular filtration rate (eGFR, continuous) and BMI (continuous).

* *p* < 0.05.

**Table 5:** HR and 95% CI for the association between PUFAs intake and HUA in males and females^†^^‡^

	Quintile of proportion of energy intake from fats					*p*-trend
	Q1	Q2	Q3	Q4	Q5	
Male						
PUFAs / %E M (Q_1_, Q_3_)	2.86 (1.99, 3.45)	4.95 (4.53, 5.47)	6.70 (6.29, 7.23)	8.73 (8.16, 9.43)	12.3 (11.3,14.7)	
Model 1^§^	1 (ref)	1.19 (0.70, 2.01)	0.90 (0.52, 1.56)	1.05 (0.62, 1.78)	1.61 (0.98, 2.66)	0.082
Model 2^¶^	1 (ref)	1.17 (0.69, 1.97)	0.91 (0.52, 1.59)	1.07 (0.63, 1.81)	1.63 (0.99, 2.70)	0.067
Model 3^††^	1 (ref)	1.20 (0.71, 2.02)	0.85 (0.49, 1.48)	1.03 (0.61, 1.76)	1.52 (0.92, 2.51)	0.131
Female						
PUFAs / %E M (Q_1_, Q_3_)	2.91 (2.15, 3.58)	5.15 (4.62, 5.54)	6.88 (6.45, 7.38)	9.19 (8.51, 9.82)	12.8 (11.5, 15.2)	
Model 1^§^	1 (ref)	0.61 (0.36, 1.03)	0.36* (0.19, 0.67)	0.76 (0.46, 1.26)	0.95 (0.58, 1.55)	0.723
Model 2^¶^	1 (ref)	0.61 (0.36, 1.04)	0.36* (0.19, 0.68)	0.79 (0.48, 1.32)	0.95 (0.57, 1.55)	0.686
Model 3^††^	1 (ref)	0.67 (0.39, 1.16)	0.41* (0.22, 0.78)	0.75 (0.45, 1.26)	0.94 (0.57, 1.55)	0.873

HUA, hyperuricemia; PUFAs, polyunsaturated fatty acids.

† Continuous variables were reported as median (Q1, Q3).

‡ The Cox proportional hazards model was used to estimate hazard ratios.

§ Model 1 adjusted for age, education, per-capita household income, geographic region, and place of residence.

¶ Model 2 additionally adjusted for physical activity level, total energy intake (continuous), and total purine intake (continuous).

†† Model 3 further adjusted for estimated glomerular filtration rate (eGFR, continuous) and BMI (continuous).

* *p* < 0.05

Dose–response relationship between proportion of energy intake from fats, dietary fatty acids intake, and HUA

We applied restricted cubic splines to model and visualize the association between the proportion of energy intake from fats, SFAs, MUFAs, and PUFAs and HUA, separately for men and women. The reference point is zero intake of fats, SFAs, MUFAs, and PUFAs, using four spline points for the respective proportions of energy intake across the distribution.

**Figure 1:**
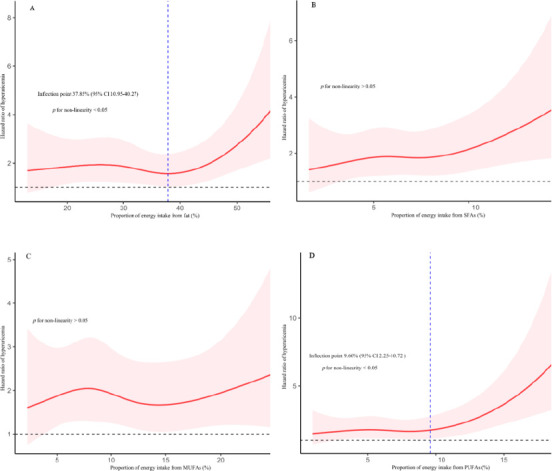
Restricted cubic spline of the association between proportion of energy intake from fats, SFAs, MUFAs, PUFAs, and HUA in men. HR is represented by solid lines, with 95% CI shown by shaded areas

A significant non-linear (J-shaped) association was found between total fat intake and HUA risk in men (*p* for non-linearity < 0.05). The HR remained stable at lower in-takes but increased significantly after an inflection point at 37.9% of total energy (95% CI 11.0–40.3) (Figure 1A). In contrast, no significant non-linear association was detected for total fat intake in women (*p* for non-linearity > 0.05).

**Figure 2:**
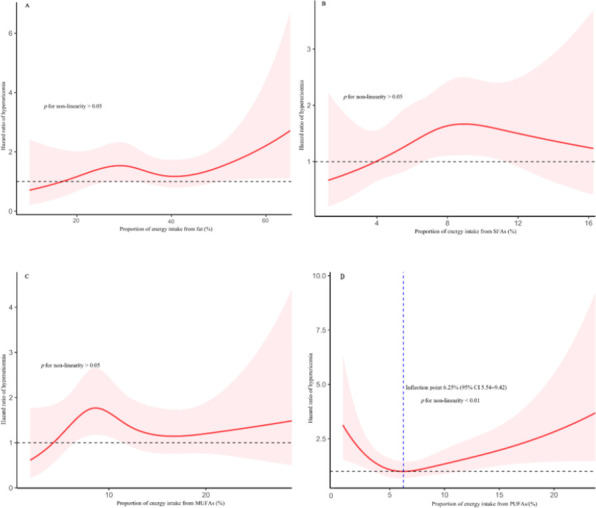
Restricted cubic spline of association between proportion of energy intake from fats, SFAs, MUFAs, PUFAs, and HUA in women. HR is represented by solid lines, with 95% CI shown by shaded areas.

The analysis of PUFAs intake also revealed distinct, non-linear patterns. Among men, a U-shaped association was observed (*p* for non-linearity < 0.05), with the nadir of risk occur-ring at a PUFAs intake of 9.60% of total energy (95% CI 2.25–10.7) (Figure 1D). For women, the relationship was L-shaped (*p* for non-linearity < 0.01); risk decreased steeply until an inflection point at 6.25% of total energy (95% CI 5.54–9.42), after which the curve plateaued (Figure 2D). Finally, we did not find a significant non-linear association with HUA risk for the proportion of energy from SFAs and MUFAs in either men or women (*p* for non-linearity > 0.05).

## DISCUSSION

In this prospective cohort study, involving 2,722 individuals with a mean follow‑up period of 6.05 years, we investigated the relationship between dietary fat energy ratio, various fatty acids, and the risk of HUA. No significant linear association was observed between dietary fat energy ratio and HUA risk in either men or women; however, RCS analysis indicated a significant J‑shaped non‑linear relationship in men. PUFAs intake in men showed a U‑shaped association with HUA risk. Among women, SFAs and MUFAs were positively associated with HUA, whereas PUFAs showed an inverse relationship; RCS indicated an L-shaped relationship between PUFAs intake and HUA.

We identified a significant J-shaped association between total fat energy ratio and the risk of incident HUA in men, an association not observed in women. This finding suggests a sex-specific, non-linear relationship where high dietary fat intake may elevate HUA risk. The biological mechanisms may be twofold: chronic high-fat intake promotes insulin resistance,^32^ which is thought to impair renal uric acid excretion by down-regulating critical urate trans-porters like URAT1 and GLUT9,^33^ and elevated circulating free fatty acids may directly in-crease uric acid production by stimulating hepatic xanthine oxidase activity through oxidative stress pathways.^15,34^ Our finding in men was consistent with that observed in other Asian populations,^35^ but it diverges from the null associations for total fat reported in the National Health and Nutrition Examination Survey (NHANES).^36^ The different outcomes likely reflect underlying differences in the primary food sources and fatty acid composition of dietary fat—such as the predominance of cooking oils and pork fat in Asian diets compared with dairy and plant oils in Western diets—as well as the overall dietary pattern in which the fat is consumed. The absence of an association in women. This sex-specific protection is likely multifactorial, but a primary contributor is the metabolic effect of estrogen, which enhances renal urate excretion and confers greater insulin sensitivity.^37,38^ Collectively, our prospective findings suggest that for men in this population, maintaining a moderate fat intake (e.g., 20–30% of energy) may be an important strategy for HUA prevention. In the context of rapid nutrition transition in China—energy-dense, high-fat diets—these findings highlight the importance of balanced fat consumption and sex-specific considerations in dietary guidance.

We observed a sex-specific association between SFAs intake and HUA. After multivariable adjustment, women in the fourth quintile of SFAs consumption had a significantly higher risk of HUA compared to those in the lowest quintile. This association was not observed in men and did not persist into the highest quintile for women. This non-linear pattern suggests a potential threshold effect, or it may reflect residual confounding among the highest consumers. The observed sex difference may arise from hormonal and metabolic factors. Diets high in SFAs are known to promote insulin resistance, oxidative stress, and hepatic lipotoxicity,^39^ processes that can elevate SUA by reducing renal urate clearance and increasing xanthine oxidase activity.^33^ Moreover, SFAs can stimulate the Toll-like receptor 4 (TLR4)-mediated inflammatory pathway, which is a central driver of metabolic inflammation.^40^ This persistent, low-grade inflammatory state may impair renal urate handling.^41^ We propose that estrogen’s improved insulin sensitivity and uricosuric effects may attenuate SFA-induced insults in women.^37,38^ Consistent with this, the DASH diet is a low-SFAs diet pattern that is associated with lower blood uric acid levels.^42^ These results highlight the importance of dietary fat quality. For women, reducing SFAs consumption from sources like processed meats and animal fats may be a targeted strategy for HUA prevention.

We observed a non-linear association between MUFAs intake and the risk of incident HUA exclusively in women, characterized by a slightly elevated hazard ratio in the third quintile. The absence of a clear dose-response and an association in men. Therefore, these metabolic impacts appear to be highly conditional, modulated by the overall dietary matrix, food sources, and sex-specific physiology. Epidemiologic studies rooted in Mediterranean populations, where extra virgin olive oil is the principal MUFAs source,^43^ consistently report an inverse association with serum uric acid.^44^ This protective signal is likely attributable not only to oleic acid but also to the rich milieu of co-consumed antioxidant polyphenols present in unrefined plant oils.^45^ In our study, MUFAs are primarily derived from animal fats derived from pork and refined vegetable oils.^46^ These sources mean that MUFAs are often consumed alongside high quantities of SFAs and are devoid of the phytochemicals found in their unrefined plant-based counterparts. This co-linearity with metabolically detrimental fats likely masks or counteracts any potential benefits of MUFAs intake. From a mechanistic standpoint, the isolated effects of MUFAs are theoretically protective. By improving insulin sensitivity,^47^ they can help mitigate the hyperinsulinemia that is known to impair renal urate excretion. However, this benefit may be conditional. The non-linear “hump” in risk observed in women may reflect a metabolic tipping point: at lower intakes, MUFAs may be beneficial, but as total fat and energy intake rise, they can still contribute to ectopic fat deposition and a pro-inflammatory state, overwhelming any subtle benefits and creating an environment conducive to HUA. The association among women in our study may reflect com-plex interactions between dietary fat quality and hormonal status, particularly across the menopausal transition when this estrogen-mediated protection wanes and susceptibility to metabolic dysfunction increases over the follow-up period.

PUFAs intake showed a non‑linear, sex‑specific association with HUA in this prospective cohort. Among men, the risk curve was U‑shaped, indicating the lowest incidence at moderate intake levels. Among women, moderate PUFAs consumption was associated with a re-duced risk, corresponding to the third quintile, beyond which the curve plateaued. Previous research examining this association has produced heterogeneous results, with some studies reporting no clear relationship between total PUFAs intake and SUA or gout and others suggesting modest inverse trends when n‑3‑rich sources—such as fish—predominate.^12, 48–51^ Such discrepancies likely reflect population‑specific differences in dietary composition, PUFAs subtype distribution. Moderate consumption of n‑3 PUFAs can enhance endothelial function,^52^ suppress inflammation,^53^ and decrease oxidative stress.^54^ These mechanisms may favor urate excretion and improve insulin sensitivity. In contrast, excessive PUFAs intake dominated by n‑6 fatty acids can promote lipid peroxidation and the formation of reactive oxygen species,^55,56^ impairing renal function and contributing to metabolic oxidative stress. The observed sex difference may be due to the role of estrogen in uric acid metabolism. Estrogen promotes renal urate clearance and attenuates inflammation.^37,57^ In men, without this hormonal influence, PUFAs effects may depend more on dietary fat quality, oxidative exposure, and insulin resistance pathways. From a public‑health perspective, dietary guidelines should prioritize improvements in both the quality and balance of dietary fats, encouraging the intake of n‑3‑rich foods such as fish, soy, and nuts.

The CHNS serves as a well-established prospective cohort study that follows data across two generations, providing a diverse and representative study population.^24,25^ Strengths of this work include its longitudinal design, large sample size, and the stability and detailed characterization of participants.^58^ Each survey wave within the CHNS adhered to standardized protocols with consistent questionnaires and methodologies, ensuring comparability of data across survey cycles. This study is the first prospective investigation to assess the association between the proportion of dietary energy derived from fats, specific dietary fatty acid intake, and the risk of HUA among healthy Chinese adults. Moreover, restricted cubic spline (RCS) analysis was applied to delineate potential dose–response relationships between dietary fat intake, fatty acid subtypes, and HUA risk. Nonetheless, several limitations should be acknowledged. First, dietary information was collected using consecutive 3-day, 24-hour dietary recalls, which may not fully reflect long term dietary habits owing to day to day variability in fat intake. This random variation could lead to exposure misclassification and attenuate true associations. Second, SUA concentrations were measured only once, precluding the evaluation of intra individual variability over time. Third, due to limited clinical data, participants with gout could not be excluded. Although likely few in number,^59^ the potential use of urate lowering therapy or diuretics in this subgroup may have influenced the observed associations. Fourth, a Cox proportional hazards model was adopted because only two follow up waves were available after baseline. While appropriate for time to event analysis under these conditions, other models might be more efficient once additional survey waves (e.g., the 2022–2024 CHNS) become available, which we plan to incorporate in future analyses. Finally, residual confounding cannot be fully excluded because factors such as family history of HUA were not collected in the dataset.

### Conclusions

In this study, we found non-linear, sex-specific associations between total and individual fatty acid intake and HUA risk. Among men, total fat as a proportion of energy intake exhibited a J-shaped relationship with HUA risk, rising noticeably above an energy ratio of about 38%, while PUFAs intake followed a U-shaped pattern with the lowest risk at moderate levels. In women, fat quality was more influential: SFAs intake increased risk, whereas moderate PUFAs intake was protective. Men should consider moderating total fat intake, while women should limit SFAs and moderately increase PUFAs to help reduce HUA risk.

## ACKNOWLEDGEMENTS

The authors express their gratitude to the individuals who took part in the survey, as well as to the teams and personnel who collaborated on the China Health and Nutrition Survey (CHNS).

## CONFLICT OF INTEREST AND FUNDING DISCLOSURES

The authors have no conflicts of interest to declare.

This study was supported by the Study of Diet and Nutrition Assessment and Intervention Technology, National Key R&D Program (No.2020YFC2006300), and Public Health Emergency Project, National Financial Projects (No.131031107000210002). The data of this study are from CHNS, which was funded by NINH, China CDC; National financial projects (No.13103110700015005); the National Institutes of Health (R01-HD30880, R01-HD38700, R24-HD050924, DK056350); and the University of North Carolina at Chapel Hill (UNC-CH) (5 R24 HD050924).

## Supplementary Material

Supplementary data
