# Supplementary material for: Association between dietary fat intake and fatty acid profiles and hyperuricemia among Chinese adults: Results from the China Health and Nutrition Survey
Source: Asia Pac J Clin Nutr. 2026 Mar 17;35(2):244–54. doi: 10.6133/apjcn.202604_35(2).0007 (PMC13012835; doi:10.6133/apjcn.202604_35(2).0007)
Supplement: Supplementary file 1 — Supplementary data [file apjcn-0035-0244-s01.pdf]

Supplementary Materials

Association between dietary fat intake and fatty acid profiles and hyperuricemia among Chinese adults: Results from the China Health and Nutrition Survey

Pengfeng Qu PhD[1], Yingying Jiao MM[1,2], Liusen Wang MM[1,3], Weiyi Li MPH[1,3], Hongru Jiang PhD[1,3], Jiguo Zhang MD[1,3], Huijun Wang MD[1,3], Bing Zhang MD[1,3], Junhua Han MD[4], Aidong Liu MD[1,3], Zhihong Wang MD[1,3]

[1]*Office of National Nutrition Plan, National Institute for Nutrition and Health, Chinese Center for Disease Control and Prevention, Beijing, China*
[2]*Hancheng Center for Disease Control and Prevention, Shaanxi, China*
[3]*Key Laboratory of Trace Elements and Nutrition, National Health Commission, Beijing, China*
[4]*Chinese Nutrition Society, Beijing, China*

Corresponding Author:
Dr Zhihong Wang, National Institute for Nutrition and Health, Chinese Center for Disease Control and Prevention, 27 Nanwei Road, Beijing 100050, China.
Tel: +86-10-6623-7008.
Email: wangzh@ninh.chinacdc.cn
Dr Aidong Liu, National Institute for Nutrition and Health, Chinese Center for Disease Control and Prevention, 27 Nanwei Road, Beijing 100050, China.
Tel: +86-10-6623-7006.
Email: liuad@ninh.chinacdc.cn
Manuscript received 10 October 2025. Initial review completed 03 November 2025. Revision accepted 04 December 2025.
doi: 10.6133/apjcn.202604_35(2).0007

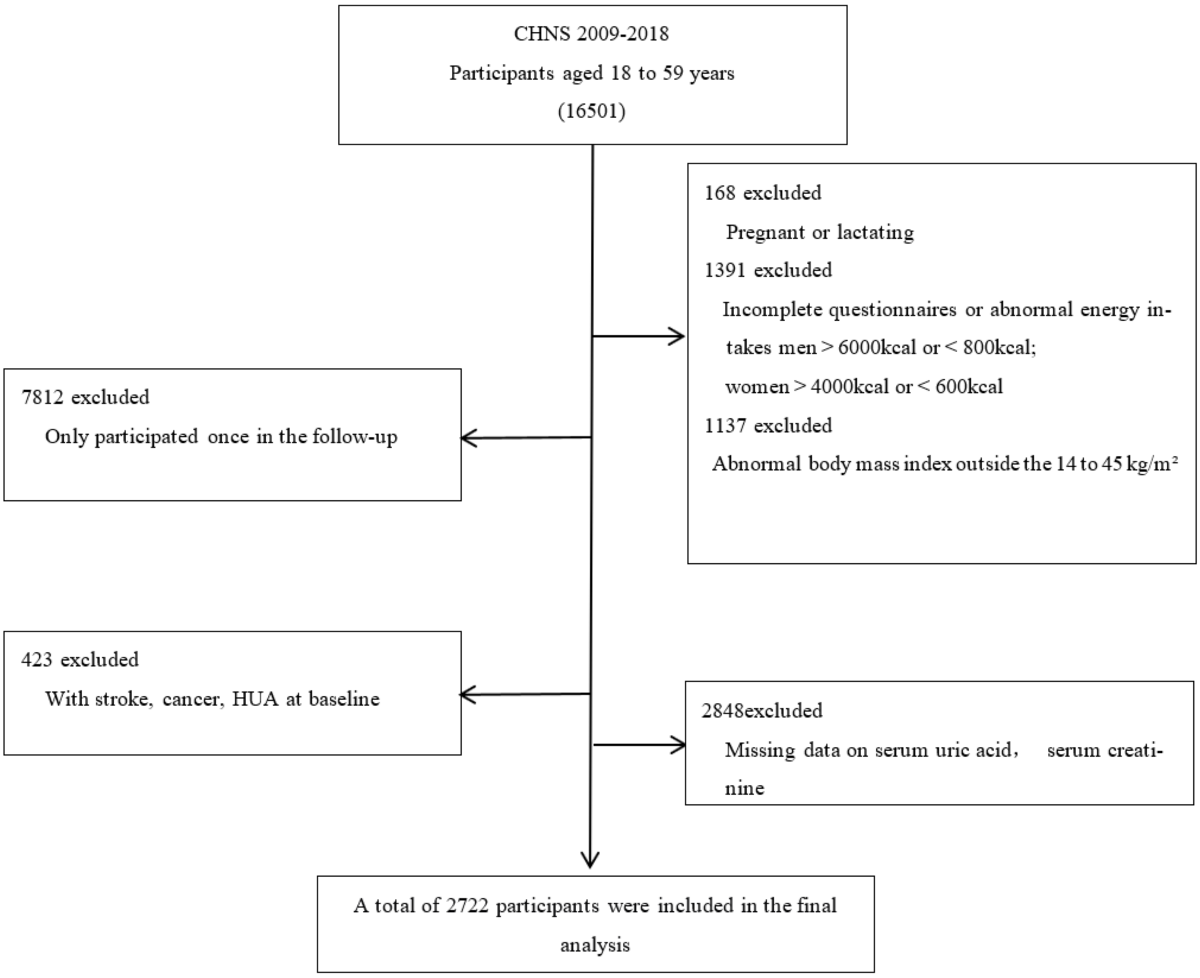

Supplementary Figure 1. Flow chart of study population selection
